# Impaired lymphatic drainage in a child with hydrops fetalis and a novel variant in the PROX1 gene: case report and review of literature

**DOI:** 10.3389/fcvm.2026.1760172

**Published:** 2026-07-29

**Authors:** Domenico Umberto De Rose, Flaminia Pugnaloni, Simonetta Costa, Nicoletta Menzella, Elisa Pisaneschi, Andrea Diociaiuti, Gian Luigi Natali, Aurelio Secinaro, Laura Massella, Maria Cristina Digilio, Francesca Campi, Antonio Novelli, Andrea Conforti, Irma Capolupo, Giovanni Vento, Andrea Dotta

**Affiliations:** 1 Neonatal Intensive Care Unit, “Bambino Gesù” Children's Hospital IRCCS, Rome, Italy; 2 Neonatal Intensive Care Unit, Policlinico Casilino, Rome, Italy; 3 Neonatal Intensive Care Unit, Fondazione Policlinico Universitario “A. Gemelli” IRCCS, Rome, Italy; 4 Translational Cytogenomics Unit, “Bambino Gesù” Children's Hospital, IRCCS, Rome, Italy; 5 Dermatology Unit and Genodermatosis Unit, “Bambino Gesù” Children's Hospital, IRCCS, Rome, Italy; 6 Interventional Radiology Unit, “Bambino Gesù” Children's Hospital, IRCCS, Rome, Italy; 7 Advanced Cardiovascular Imaging Unit, “Bambino Gesù” Children's Hospital, IRCCS, Rome, Italy; 8 Pediatric Nephrology Unit, “Bambino Gesù” Children's Hospital, IRCCS, Rome, Italy; 9 Medical Genetics Unit, “Bambino Gesù” Children's Hospital, IRCCS, Rome, Italy; 10 UniCamillus-Saint Camillus International University of Health Sciences, Rome, Italy; 11 Neonatal Surgery Unit, “Bambino Gesù” Children's Hospital, IRCCS, Rome, Italy

**Keywords:** chylothorax, congenital anomalies, neonate, newborn, peritoneal dialysis, preterm

## Abstract

PROX1 is a gene that encodes a protein that may play a key role in the development of the lymphatic system. This report describes impaired lymphatic drainage, with non-immune hydrops fetalis, congenital bilateral chylothorax, chylous ascites, and the need for peritoneal dialysis in a preterm neonate born at 32 weeks of gestation. The child died at about seven months of life. Clinical Exome Sequencing revealed the novel heterozygous variant c.502C > T in the PROX1 gene, which determines the change p.Arg168Trp at the protein level. This *de novo* missense variant was not detected in the analyzed parental DNA samples and has not been previously described in the literature. Although the identified PROX1 variant represents a biologically plausible contributor to the phenotype, causality cannot currently be established, and additional environmental or maternal factors cannot be excluded. Identifying this variant in affected individuals can have implications for perinatal and postnatal management and genetic counseling. To the best of our knowledge, this is the first case reported of a child carrier of a PROX1 variant with hydrops fetalis. PROX1 screening could be requested in prenatal diagnosis of hydrops fetalis and included in Next-Generation DNA Sequencing panels.

## 1 Introduction

The term hydrops fetalis (HF) describes excessive, pathologic fluid accumulation within the fetal soft tissues and body cavities and can have an immune (IHF) or a non-immune etiology (NIHF, when not caused by red cell alloimmunization). The incidence of NIHF in live-born infants is reported to be 1 per 4,000. Four main mechanisms have been proposed to explain the distribution of body fluids in NIHF: (1) increase in hydrostatic capillary pressure; (2) reduction in plasma oncotic pressure; (3) obstruction of lymphatic flow; and (4) damage to peripheral capillary integrity. NIHF may result from 1 or more of these mechanisms depending on the cause (1).

Greater than one-third (37%) of cases of NIHF with negative clinical workup for anemia, infections, and chromosomal disorders have a monogenic disorder detectable by exome sequencing (2). RASopathies are the most common set of monogenic syndromes identified by exome sequencing for NIHF (3), but causes include neuromuscular, metabolic, lymphatic, cardiovascular, or other anomalies (2). Increasing evidence suggests that genes involved in lymphatic development and central conducting lymphatic anomalies, including PIEZO1, EPHB4, FLT4 and CCBE1, may contribute to NIHF (4–7).

Thanks to the development of next-generation sequencing approaches, the knowledge about the genetic background of NIHF is increasing. PROX1 is a gene that encodes a transcription factor with a key role in developing several organ systems, including the lymphatic system development (8–10).

We here report a case involving a preterm neonate carrier of a novel PROX1 variant, with non-immune hydrops fetalis, congenital bilateral chylothorax, chylous ascites, and the need for peritoneal dialysis. To our knowledge, this is the first case report on this association.

## 2 Case report

### 2.1 Clinical presentation

A 2,250 grams female infant was born at 32 weeks gestation by cesarean delivery because of bilateral fetal hydrothorax and maternal HIV infection. Apgar scores were at 5 at 1 min and 7 at 5 min. At birth, the infant, who appeared hydropic with oedema of the trunk, head, limbs, and pitting sign, required orotracheal intubation for cardiorespiratory depression.

During pregnancy, the mother had been on antiretroviral therapy with Raltegravir and Emtricitabine/Tenofovir disoproxil, on diet therapy for gestational diabetes, and on levothyroxine for hypothyroidism. Moreover, the mother had a smoking habit.

The diagnosis of bilateral fetal hydrothorax was made at 28 weeks gestation. Intrauterine thoracentesis was performed at 30 weeks, and a thoraco-amniotic shunt was placed after a second fetal thoracentesis at 32 weeks.

Immediately after birth, the infant presented respiratory distress syndrome, requiring surfactant administration; the presence of bilateral chylothorax led to respiratory failure, requiring bilateral pleural drain placement.

The chylothorax was treated with fasting and octreotide administration, obtaining a clear reduction in chylous production, such as allowing the removal of the chest drains at 30 days of life.

However, it was never possible to feed the baby because every time the baby was fed with special low-fat milk, the chylothorax relapsed. Chylothorax was associated with chylous ascites, which required four paracentesis and subsequent permanent abdominal drainage placement. Propranolol was also added to the octreotide, without benefit.

**Figure 1:**
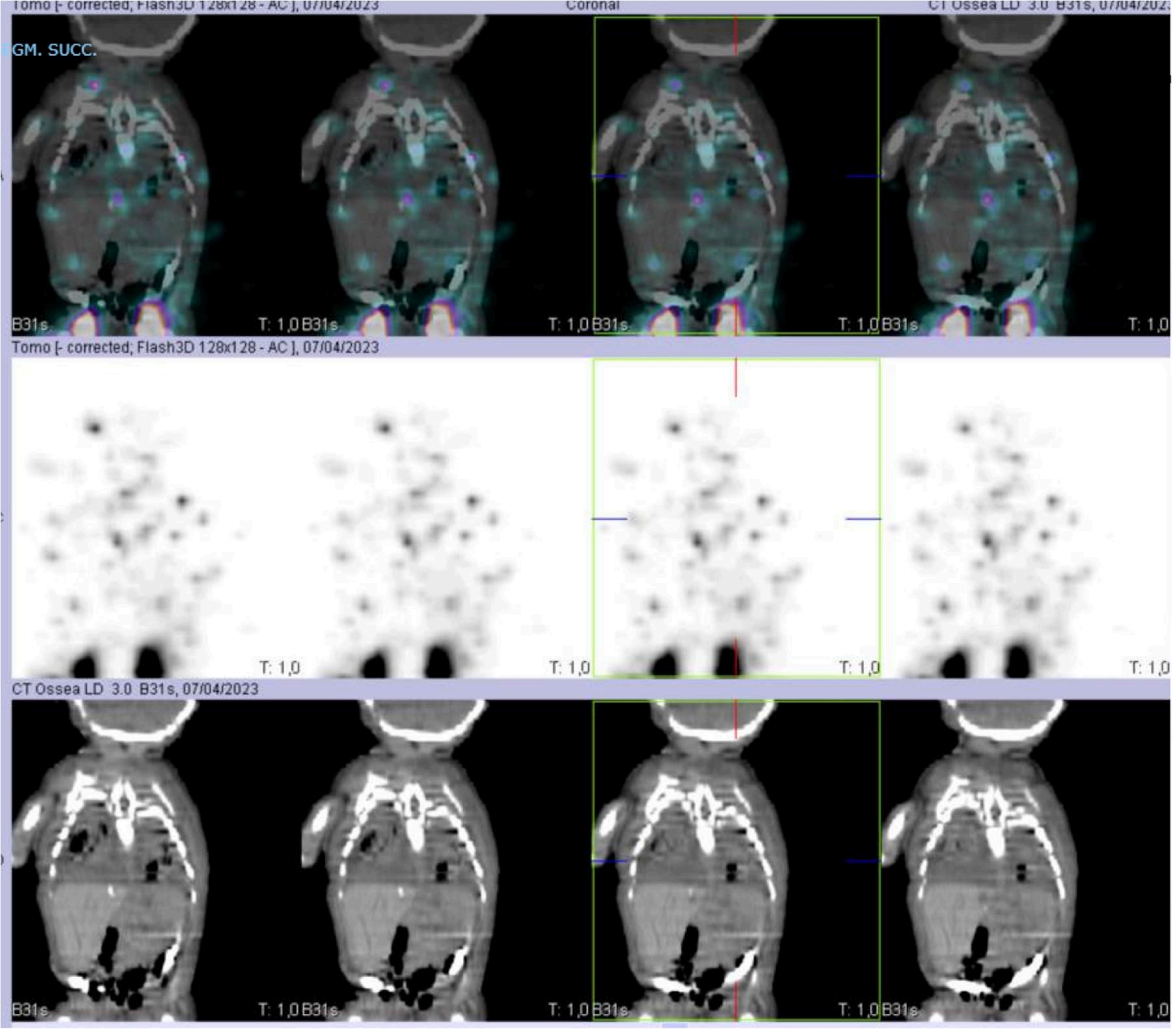
Lymphoscintigraphy performed at approximately 3 months of life. Bilateral transit of the radiopharmaceutical tracer was documented up to the thoracic duct, visualized in its thoracic portion. A minimal amount of tracer was detected at the level of the known pleural effusion, particularly in the left basal and bilateral apical regions. No pathological tracer accumulation was observed in the abdominal compartment at standard imaging timepoints. However, approximately 20 h after tracer administration, lymphatic fluid was aspirated from the abdominal drainage catheter and analyzed by gamma-counter; two 2 mL samples demonstrated significant radioactivity counts (841 and 791 cpm), indicating that the chylous ascites originated from thoracic lymphatic leakage rather than from an independent abdominal source.

At three months of life, the infant underwent scintigraphy of the lymphatic system which documented the presence of leakage at the level of the thoracic duct, but not at the abdominal level. Twenty hours after tracer injection, the radiopharmaceutical tracer was unexpectedly recovered from the abdominal drainage catheter. This finding indicated that the tracer, after leaking from the thoracic duct into the pleural space, had migrated caudally into the peritoneal cavity, suggesting that the chylous ascites was a downstream consequence of central lymphatic leakage rather than an independent abdominal source. Sequential scintigraphic images documenting tracer distribution over time are shown in Figure 1.

**Figure 2:**
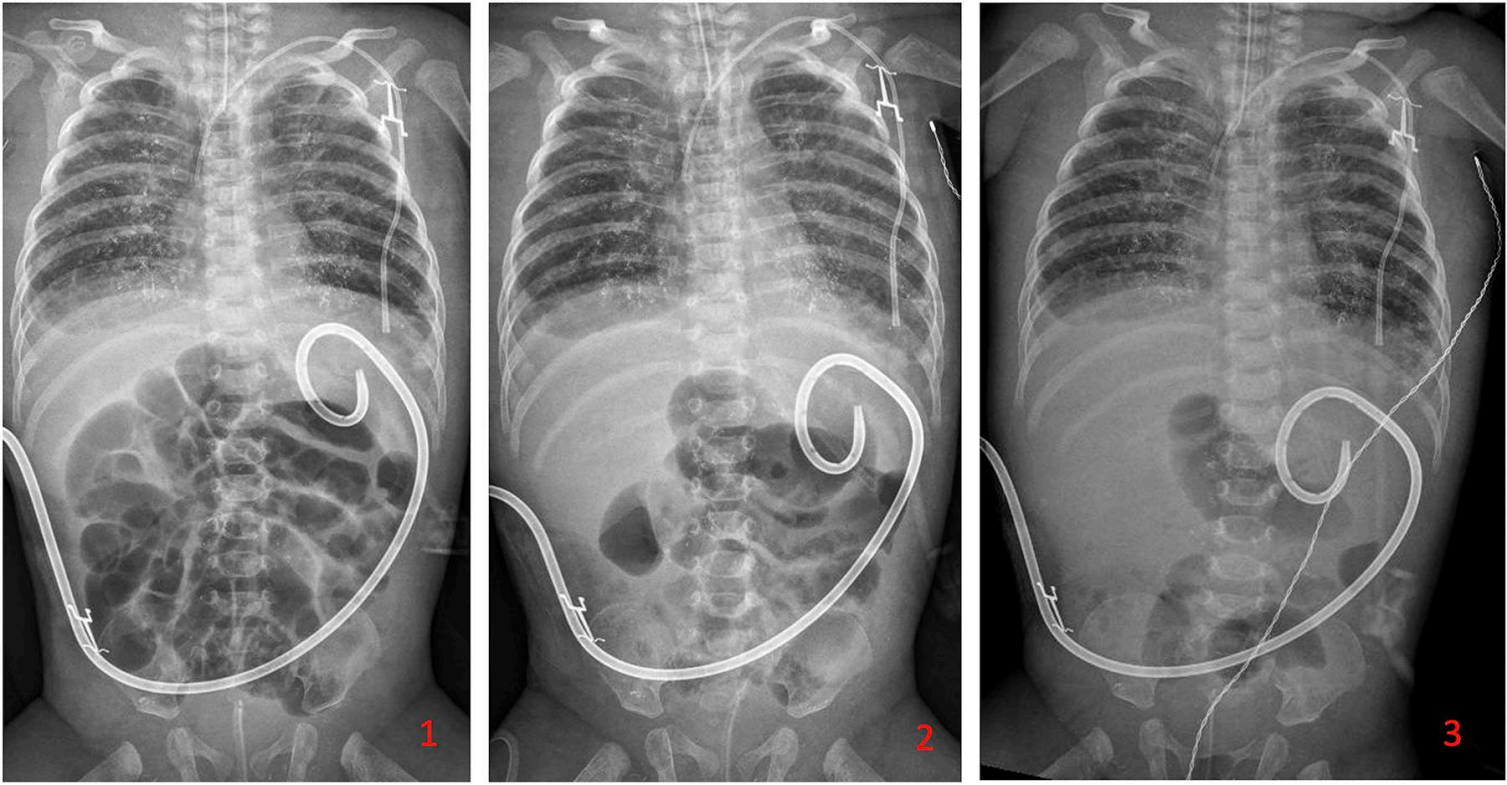
Serial radiographic imaging following attempted thoracic duct sclerotization. Fluoroscopic images obtained 1–2 and 4 days after the interventional radiology procedure demonstrate persistent failure to opacify the thoracic duct, confirming the unsuccessful outcome of the sclerotization attempt.

At 5 months of life, in consideration of the non-responsiveness to medical treatment, an attempt at sclerotization of the thoracic duct was performed, using an interventional radiology procedure. It was unfortunately unsuccessful since, at subsequent serial radiographic checks, opacification of the thoracic duct was not achieved (Figure 2).

The postnatal clinical course was complicated by renal intraparenchymal vasoconstriction with oliguria. Renal ultrasound performed on the day of birth showed normally sized kidneys with mild cortical hyperechogenicity; color Doppler evaluation of intraparenchymal vessels demonstrated increased peak systolic velocity with reduced diastolic flow in multiple samplings, consistent with elevated intraparenchymal resistance indices, suggesting early renal vasoconstriction. This condition was initially treated with fenoldopam. This condition resulted, at 25 days of life, in acute renal failure following septic shock from *Staphylococcus epidermidis*, requiring, in addition to therapy with fenoldopam, diuretic therapy with furosemide and ethacrynic acid and, at 28 days of life, the start of the peritoneal dialysis, that was continued for thirteen days. At 2 and 5 months of life, following two other episodes of systemic infection, the infant resumed the second and third courses of peritoneal dialysis, continued for 10 and 33 days, respectively.

**Figure 3:**
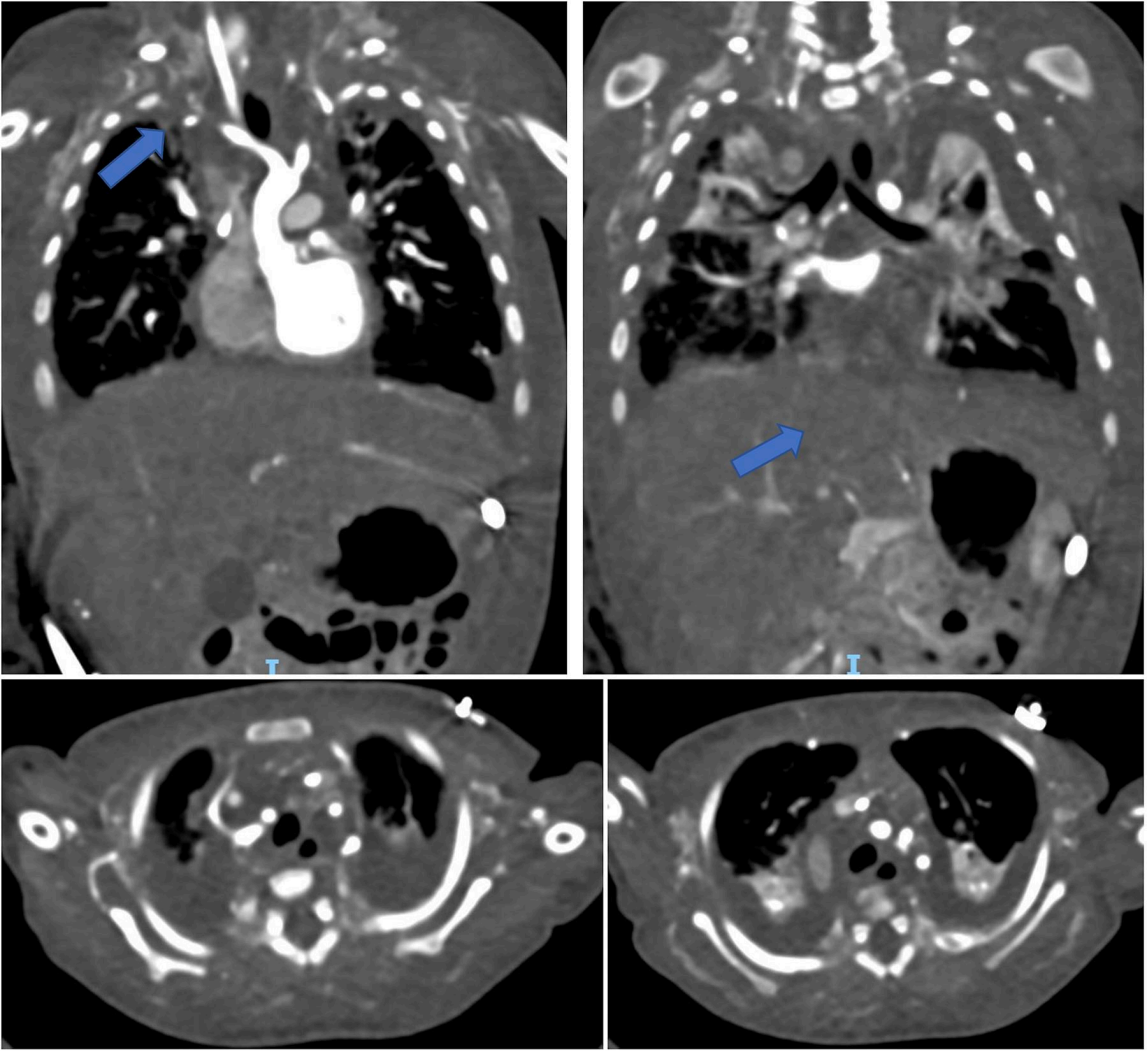
CT imaging demonstrating thrombosis of the inferior vena cava and thrombosis of the right subclavian vein.

During hospitalization, the baby presented blood coagulation complications: at 45 days of life, thrombosis of the inferior vena cava associated with caval epicutaneous catheter occurred, treated first with alteplase and then with enoxaparin; at 4 months of life thrombosis of the right subclavian vein was also diagnosed (Figure 3).

At 6 months of life, the baby was transferred to the IV-level NICU of “Bambino Gesù” Children's Hospital for further examinations and to rule out a possible surgical option.

**Figure 4:**
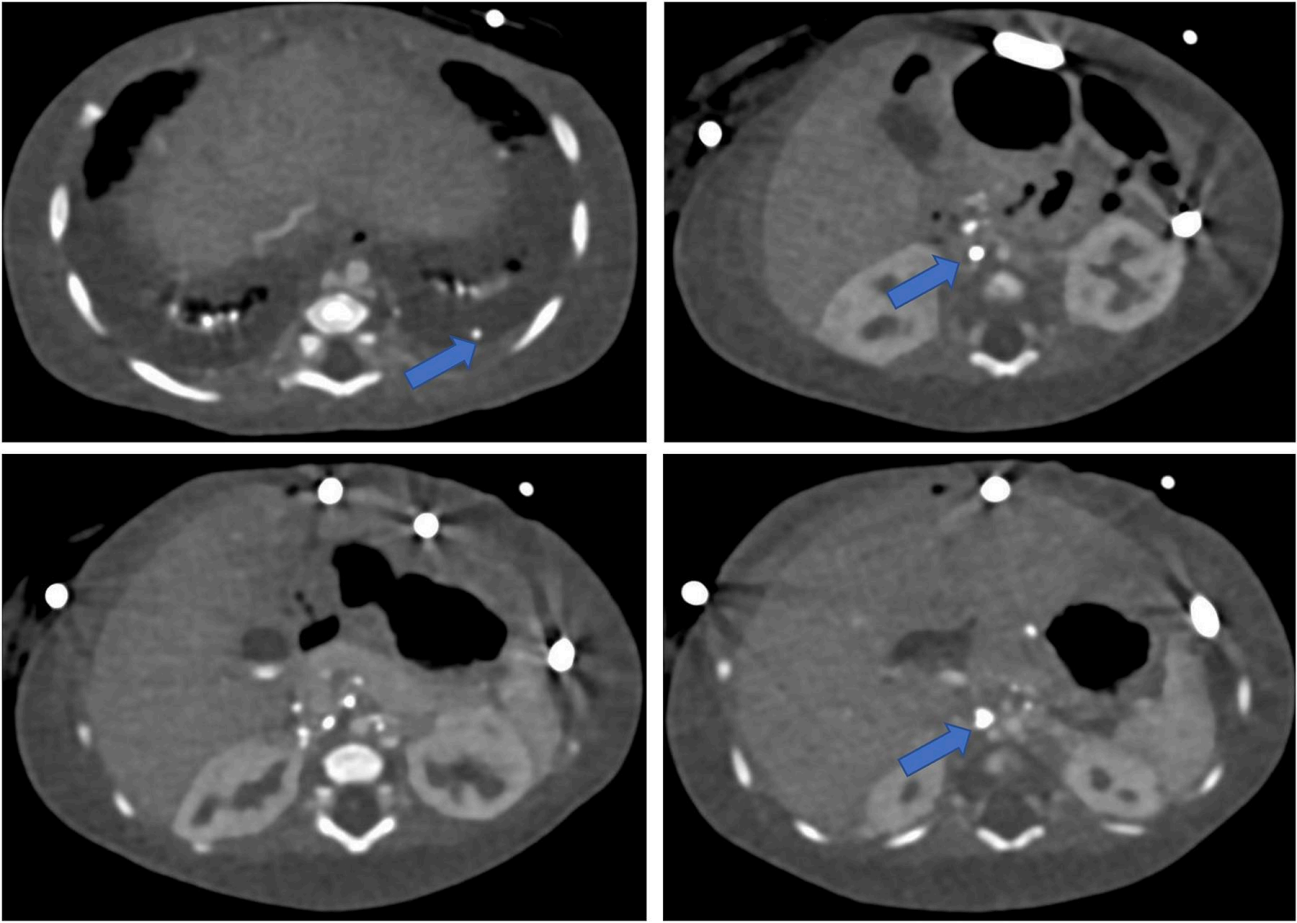
CT imaging demonstrating pleural and abdominal calcification nodules.

A further thoracoabdominal CT revealed multiple minute images with calcific density of the venous vascular district, particularly the abdominopelvic, and residuals at the posterior pleuro-parenchymal pulmonary level (Figure 4). Some calcific spots were also found in the retroperitoneal area (near the pancreas and kidneys) and at the level of the main para-aortic and celiac lymphatic stations. The pulmonary veins were patent, with thinning of the left veins at the outlet into the atrium. The inferior vena cava was completely obliterated, involving the drainage site of the renal veins, with evidence of multiple irregularities with calcific density along the course of the vessels. A compensatory dilation of the azygos-hemiazygos system and of the paravertebral circles was evident, whereas the superior caval venous axis was patent. Concerning renal drainage, renal veins were patent at the hilum with not well-delineated venous discharge, probably via lumbar collateral circulation in the azygos-hemiazygos system. The spleno-mesenteric-portal axis was also patent.

A thrombophilia diagnostic work-up was performed to rule out any coagulation anomalies (Table 1). This revealed a homozygous C677 T mutation in the MTHFR gene, with normal homocysteine levels. Coagulation screening showed a prolonged aPTT, markedly elevated D-Dimer, and reduced antithrombin activity. Factor X activity was also reduced. Von Willebrand factor antigen and ristocetin cofactor activity were markedly elevated, likely reflecting a reactive acute-phase response in the context of severe systemic illness. Protein S was within normal limits, while Protein C was mildly reduced, consistent with consumption in the setting of critical illness.

**Table 1:** Thrombophilia work-up results.

Test	Result	Reference Range
Prothrombin Time, %	83	70–125
INR	1.07	0.80–1.20
aPTT, seconds	47.4	20.0–38.0
Fibrinogen, mg/dL	286	200–400
D-Dimer, ng/mL	5,703	< 500
Antithrombin, %	61	70–140
Factor X, %	51.9	70.0–140.0
von Willebrand Factor Antigen, %	312.0	62.0–158.0
von Willebrand Factor Ristocetin Cofactor, %	297.0	70.0–140.0
Protein S Free, %	81.0	54.0–110.0
Protein C, %	67	70–140

Concurrently, the general conditions of the baby worsened again with anuria and respiratory distress: peritoneal dialysis was started again, as mechanical ventilation.

**Figure 5:**
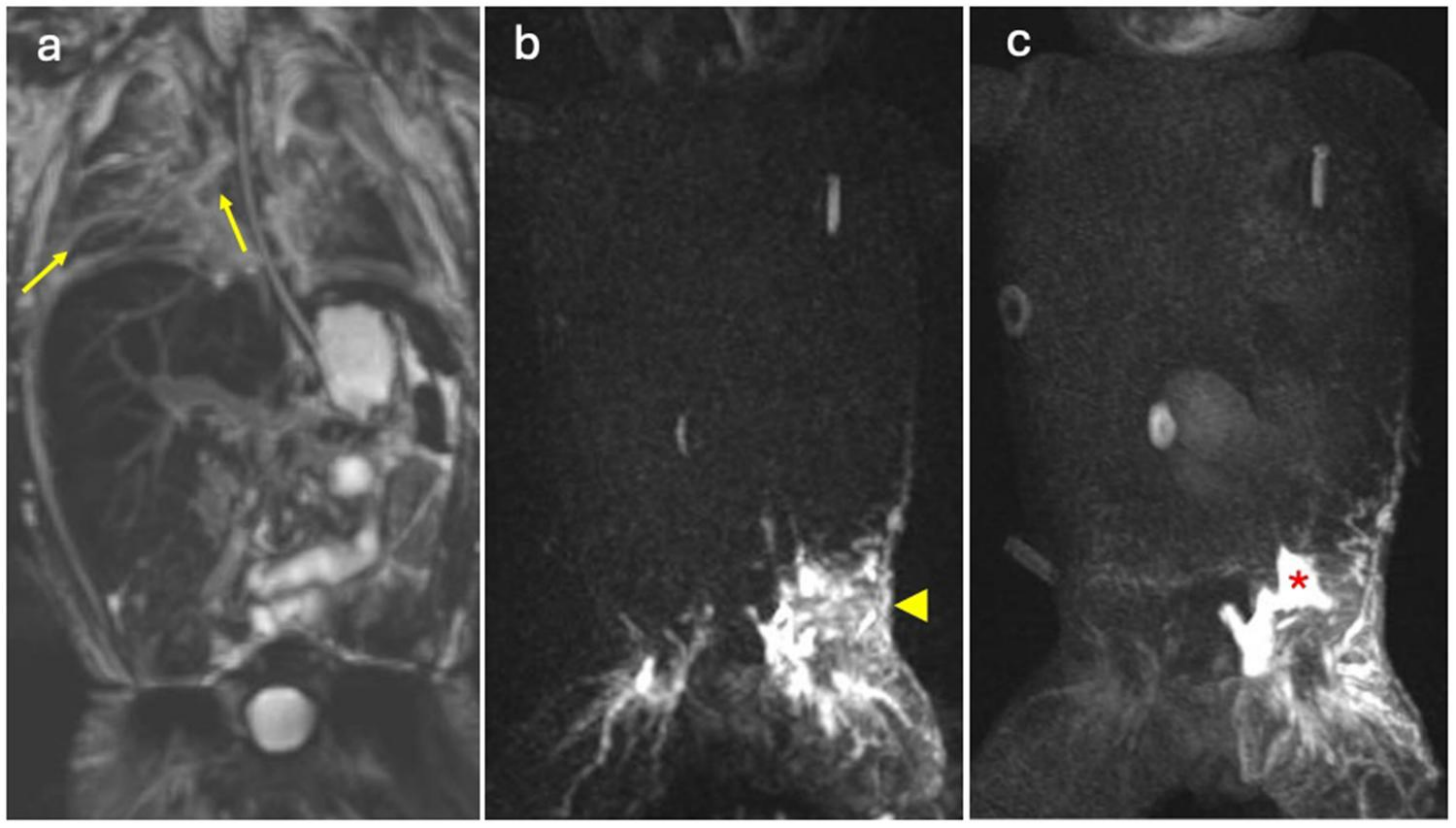
Dynamic magnetic resonance lymphangiography. Static MR lymphangiography (panel **a**) revealed a faint and diffuse T2 hyperintensity involving the perivisceral soft tissues of the retroperitoneal, mesenteric, and mediastinal regions, as well as the entire subcutaneous layer. This finding was associated with T2-hyperintense thickening of the peribronchovascular interstitium and of the subpleural septa, along with a minimal fluid-related pleural separation (yellow arrows), consistent with features of generalized lymphatic congestion. A dynamic magnetic resonance lymphangiography (panel **b** and **c**) was also performed through bilateral femoral intranodal injection of gadolinium contrast agent, demonstrating opacification of the lymphatic ducts within the iliac–femoral region, which exhibited a markedly ectatic and serpiginous morphology. The lymphatic collectors of the thoracoabdominal district were not visualized in the more cranial segments. In addition, a few ectatic lymphatic ducts of the thigh were opacified through retrograde filling, accompanied by numerous collateral lymphatic channels along the abdominal wall (panel b yellow arrowhead). In the delayed phases, the presence of contrast medium within the peritoneal cavity was documented (panel c red asterisk). Overall, these findings are consistent with a severely delayed or obstructed central lymphatic outflow, associated with peripheral lymphostasis and the development of superficial collateral pathways, as well as a possible leakage or abnormal communication between the caudal lymphatic networks and the peritoneal cavity.

A dynamic magnetic resonance lymphangiography (Figure 5) was performed, revealing the opacification of the lymphatic ducts of the iliac-femoral district with an ecstatic and serpiginous appearance and the lack of the collectors of the thoracoabdominal district more cranially. Furthermore, some ectatic lymphatic ducts of the thigh opaqued by retrograde and numerous lymphatic collateral circulations of the abdominal wall were observed. In the later phases, the passage of contrast medium at the level of the peritoneal cavity was documented. Described findings were interpreted as compatible with an extremely slowed/obstructed central lymphatic outflow with peripheral lymphostasis/superficial collateralization and doubtful leak/communication between more caudal lymphatic circles and the peritoneum.

Serial cranial ultrasounds suggested a severe picture of cerebral atrophy, confirmed by the findings of papillary pallor, filiform vessels, and dystrophic retina in the middle and extreme periphery at ophthalmological evaluation. Brain resonance magnetic imaging was not performed due to the extreme clinical instability. Clinically, the child presented a developmental delay consistent with prematurity and prolonged NICU hospitalization. She died at about seven months of life despite intensive care, due to progressive anasarca.

### 2.2 Genetic findings

Standard karyotype and Single Nucleotide Polymorphism array (SNP-array, with CytoSNP-850 K platform at an average resolution of 100 kb) did not show relevant findings. Next-generation sequencing (NGS)/Clinical Exome Sequencing (CES) was performed, using ClinEX pro kit - Clinical Exome (4bases), in the platform NovaSeq6000 (Illumina). In silico analysis in trios (proband and parents) was performed for coding regions and exon-intron junctions of the genes associated with congenital anomalies of lymphatic anomalies: ABCC9, ADAMTS3, AKT1, ANGPT2, ANTXR2, BCL2, BCL6, BRAF, CBL, CCBE1, CDC42, CDK5, CELSR1, CTNNB1, DCHS1, DNMT1, EPHB4, FAT4, FLT4, FOXC2, FZD4, GATA2, GJC2, GLA, GUSB, HLA-DRB1, HRAS, IKBKG, INPPL1, KIF11, KIF7, KRAS, LRP5, LZTR1, MAP2K1, MAP2K2, MAPK1, MRAS, NAA10, NAGA, NDP, NLRP3, NRAS, PIEZO1, PIK3CA, PMM2, PTEN, PTPN11, PTPN14, RAF1, RASA1, RASA2, RELN, RIT1, RRAS, RRAS2, SHANK3, SLC35D1, SOS1, SOS2, SOX18, SPG11, SPRED2, THSD1, TIE1, TSC1, TSC2, TSPAN12, VEGFC, XYLT2, ZNF408, RNASEH2B, BLTP1, SHOC2, KMT2D, ACTB, GRIP1, LAMB2, CACNA1C, MYH7, ENPP1, ABCC6, SCN5A, FLNA, MYBPC3, ALPK3, IDUA, GNPTAB, GALNS, SMPD1, NPC1, ASAH1, GLB1, CTSA, NEU1, SLC17A5, GBA1, SUMF1, ALG1, ALG8, ALG9, MGAT2, MVK, EBP, MRPS22, AARS2, TAFAZZIN, NDUFB10, LARS2, HADHA, HADHB, AHCY, GBE1, SGPL1, GLMN, HBA1, HBA2, SLC4A1, SPTA1, SPTB, CDAN1, SEC23B, KLF1, GATA1, PRF1, UNC13D, RPL11, RPL35A, RPL15, RPS19, G6PD, TALDO1, UROS, PKLR, GPI, PTH1R, COL2A1, SLC26A2, SLC25A4, IFT122, ITGA9, DYNC2H1, IFT80, NEK1, WDR35, KIAA0586, GLE1, RYR1, CHRNG, CHRNA1, CHRND, RAPSN, DOK7, MUSK, SMN1, SMN2, BICD2, ASCC1, NEB, TPM2, ACTA1, LMOD3, KLHL40, ERCC5, DMPK, CYP21A2, FZD6, FOXP3, ADA2, AGGF1, BSND, CALCRL, CARS2, CASP10, COL11A1, COL1A1, COL1A2, DYNC2I1, DYNC2I2, EPB41, FAS, FASLG, FIG4, FLNB, FOXF1, GATB, GATC, GYPC, HSPG2, KIF20A, MCM10, MECOM, MMACHC, NEK9, NR1H4, NSF, PIGA, PLD1, PPP3CA, PRKCD, QRSL1, RASGRP1, RHD, RPL18, RPL26, RPL27, RPL31, RPL35, RPL5, RPS10, RPS15A, RPS17, RPS20, RPS24, RPS26, RPS27, RPS28, RPS29, RPS7, TAPT1, TRIP11, TSR2, UROD, VAC14, WNT7A, PROX1, ACTA2, ACTC1, ACVR2B, ACVRL1, ANKRD1, B3GAT3, BMPR2, CFC1, CITED2, CREBBP, CRELD1, CHD7, DDX11, EFEMP2, EFTUD2, EHMT1, ELN, ENG, EVC, EVC2, EYA4, FBN1, FBN2, FOXH1, GATA4, GATA5, GATA6, GDF1, GJA1, GJA5, HAND2, ISL1, JAG1, KANSL1, KCNA5, KCNJ8, KCNK3, LEFTY2, MCTP2, MED12, MED13L, MFAP5, MYCN, MYH6, NF1, NKX2–5, NKX2–6, NODAL, NOTCH1, NOTCH2, NR2F2, PPP1CB, PRKG1, PRDM6, SALL1, SALL4, SMAD1, SMAD3, SMAD4, SMAD6, SMAD9, SPRED1, TAB2, TBX1, TBX20, TBX3, TBX5, TFAP2B, TGFB2, TGFB3, TGFBR1, TGFBR2, ZFPM2, ZIC3, DHCR7, KDM6A, OFD1, ARID1B, SMARCB1, ARID1A, SMARCA4, SMARCE1, RAB23, NIPBL, RAD21, SMC3, HDAC8, SMC1A, ZEB2, EP300, ARHGAP31, DLL4, DOCK6, EOGT, RBM10, RBPJ, RECQL4, CDKN1C, RPS6KA3, WDPCP, MID1, SRCAP, UBR1, SETD5, SPECC1L, NXN, CDK13, CHD4, CHD8, DNMT3A.

The reads were aligned to human genome build GRCh37. Variant calling was performed with Dragen Germline Enrichment application of BaseSpace (Illumina, San Diego, CA, USA) while variant annotation and phenotype-based prioritization of candidate genes were carried out through the Geneyx Analysis software (Geneyx Genomex). A minimum depth coverage of 30X was considered suitable for analysis. Exome sequencing data filtering was performed to identify protein-altering, rare variants (allele frequency <1% across public databases). Variant assessment was performed utilizing public population databases (gnomAD, ClinVar, Franklin, and HGMD). In silico pathogenicity predictions were systematically evaluated using CADD, REVEL, and SIFT. Variant classification followed the standard guidelines of the American College of Medical Genetics and Genomics and the Association for Molecular Pathology (ACMG/AMP) (11).

**Figure 6:**
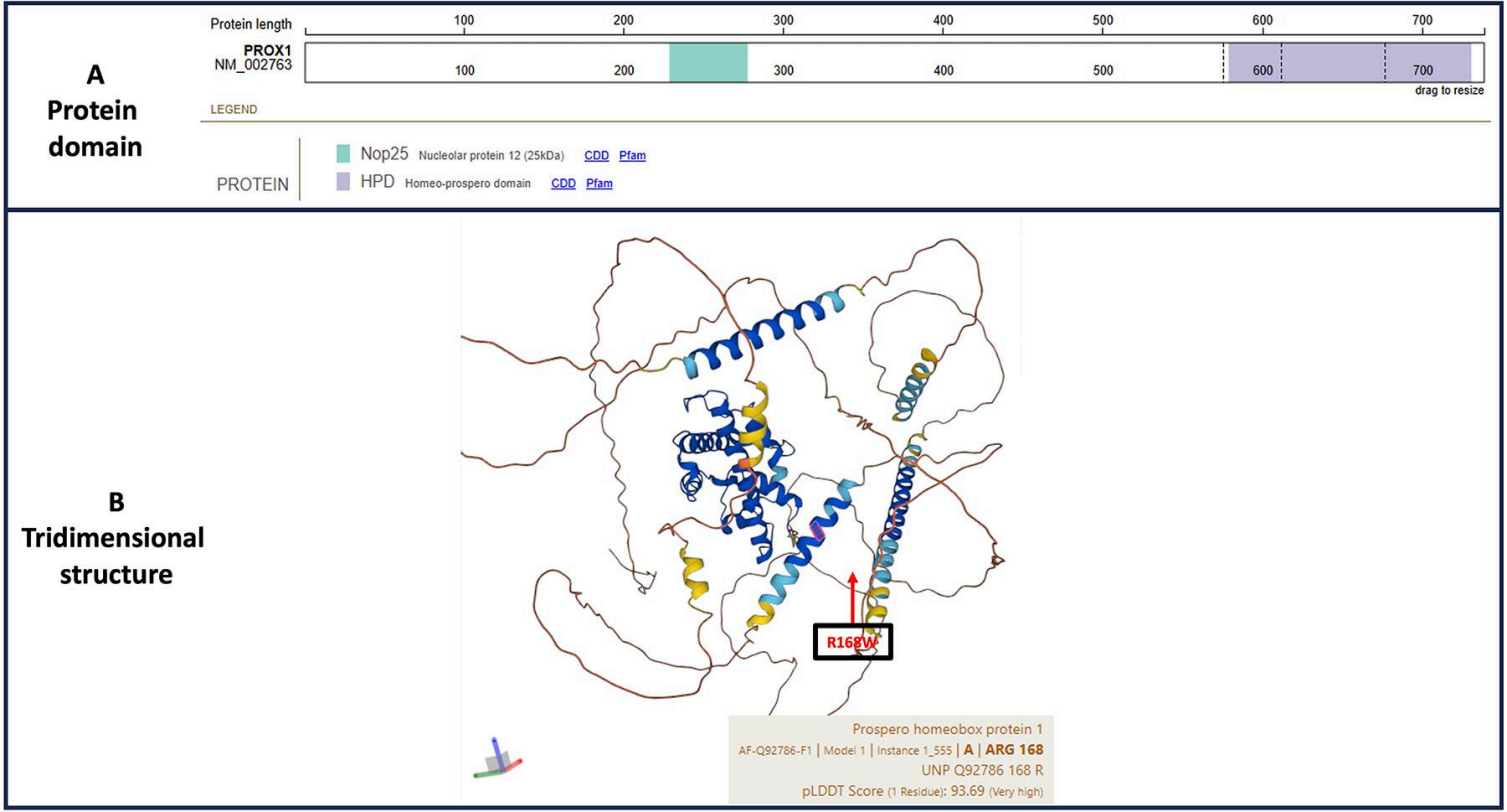
PROX1 protein domain and tridimensional structure of the p.Arg168Trp variant. Panel **A**: Schematic linear representation of the PROX1 protein (NM_002763, 737 amino acids) showing the Nop25 domain (Nucleolar protein 12, 25 kDa; teal) and the Homeo-prospero domain (HPD; purple). The p.Arg168Trp substitution is located at position 168, in a structured region proximal to the Nop25 domain. Panel **B**: AlphaFold-predicted tridimensional structure of PROX1 (AF-Q92786-F1), with the R168W mutation site indicated by a red arrow. The affected residue displays a very high local structural confidence score (pLDDT 93.69), suggesting that substitution of the positively charged arginine with the bulky hydrophobic tryptophan is likely to disrupt local protein conformation and potentially impair PROX1 function.

Clinical Exome Sequencing revealed the novel heterozygous variant c.502C > T in the PROX1 gene (NM_001270616.2), which determines the change p.Arg168Trp at the protein level (Figure 6). This *de novo* missense variant was not detected in the analyzed parental DNA samples and has not been previously described in the literature and in the gnomAD database.

Although this variant fulfilled several ACMG/AMP evidence criteria (PS2 Strong - *de novo* occurrence with confirmed maternity and paternity, in a patient with the disease and no family history; PM2 Supporting - absent from the Exome Sequencing Project, 1,000 Genomes Project, and Exome Aggregation Consortium; PP2 Supporting - gnomAD missense Z-score for PROX1 of 3.5, exceeding the threshold of 3.12, indicating intolerance to missense variation; PP3 - in silico predictors unanimously supporting a deleterious effect: CADD pathogenic strong 33, REVEL pathogenic supporting 0.76, SIFT pathogenic supporting 0), PROX1 is currently still regarded as an emerging disease gene with an incompletely established gene–disease relationship. Therefore, in the context of the present report, we consider this finding as a compelling candidate variant (variant of uncertain significance in an emerging disease gene), pending additional independent clinical observations and functional validation.

Further, two heterozygous variants of unknown significance (VUS) were found:

- the first one in the gene DOCK6 (NM_020812.4), c.3022G > A (p.Val1008Met), with maternal segregation;
- The second one in the gene NIPBL (NM_133433.4), c.5760C > G (p.His1920Gln), with paternal segregation.

## 3 Discussion

Herein, we report the case of an infant presenting with impaired lymphatic drainage, non-immune hydrops fetalis, congenital bilateral chylothorax, chylous ascites, and the need for peritoneal dialysis, occurring in association with a *de novo* heterozygous PROX1 variant.

Non-immune hydrops fetalis (NIHF) encompasses a broad etiological spectrum. Major structural causes include cardiovascular anomalies (20%–30% of cases), chromosomal abnormalities, hematologic disorders (notably alpha-thalassemia), infectious etiologies (parvovirus B19, CMV), thoracic lesions, and skeletal dysplasias. Metabolic and storage disorders, though individually rare, collectively represent a relevant proportion. Monogenic causes are increasingly recognized: more than one-third of NIHF cases with negative conventional workup harbor a detectable disorder by exome sequencing. Among these, RASopathies are the most frequent, followed by neuromuscular, metabolic, and lymphatic disorders (3). Genes involved in lymphangiogenesis (including PIEZO1, EPHB4, FLT4, and CCBE1) have been specifically implicated in NIHF through impaired lymphatic development, with generalized lymphatic dysplasia (GLD) and central conducting lymphatic anomaly (CCLA) (4–6, 12, 13).

The gene PROX1 (PROSPERO-Related Homeobox1, OMIM # 601546) encodes a homeobox transcription factor playing a crucial role during embryonic development of several tissues, including the lens, retina, liver, pancreas, heart, and lymphatic vasculature (10).

PROX1 acts as a master regulator of lymphatic endothelial cell fate and maintenance. Two major theories have been postulated to explain lymphatic system development. Mice studies largely support Florence Sabin's centrifugal sprouting theory for lymphatic vessel development. This theory states that lymphatic endothelial cells originate from embryonic veins, forming primary lymph sacs and sprouting to create a lymphatic network (14). An alternative theory by Huntington and McClure suggests these cells arise independently from mesenchymal precursors. It is unclear if mesenchymal precursors contribute similarly to mammals, though circulating lymphatic progenitor cells do participate in pathological lymphangiogenesis (15).

Using various vertebrate model organisms has demonstrated that the lymphatic system's venous origin is a well-conserved process in lymphangiogenesis across different species (16).

In mice, PROX1 is specifically expressed in lymphatic endothelial cells, which give rise to the lymphatic system by budding and sprouting. Wong et al. found in 2017 that in transgenic mice models, lymphatic endothelial cell (LEC)-specific deletion of CPT1A, a rate-controlling enzyme in fatty acid beta-oxidation, inhibits lymphatic growth (9). This has also been suggested by Geng et al., who demonstrated that the development of lymphatic valves is impaired in the PROX-1 haploinsufficient murine models, suggesting a role for this gene in lymphatic drainage (8). Together, these studies suggest that targeted inactivation of PROX1 arrests lymphatic vascular development without affecting blood vasculature.

Increasing evidence suggests that PROX1 acts as a master regulator of lymphatic endothelial cell fate and maintenance. However, recent genetic lineage-tracing experiments have revealed that non-venous lineages can also contribute to lymphatic development in peripheral organs and tissues (17).

Indeed, the downregulation of PROX-1 expression results in the loss of typical lymphatic cell junction characteristics (VE-cadherin) and the gain of blood vessel features (pericytes and platelets) (18).

Recently, Kazenwadel et al. discovered that a significant number of mice homozygous for deletion of the PROX1–11 kb enhancer died during neonatal life, with seriously damaged neonates demonstrating considerable enlargement of the jugular and thoracic regions. After embryonic day 13.5, homozygous mutant mice showed lymphatic vascular abnormalities, but those that survived for a week tended to flourish and reproduce normally. The PROX1–11 kb enhancer worked in a tissue-specific way, and PROX1 expression was decreased owing to enhancer loss, resulting in partial loss of LEC identification and reversion to blood vascular endothelial cell identity (10).

These experimental observations strongly support a role of PROX1 in lymphatic development; however, evidence linking PROX1 variants to human disease remains limited, and genotype–phenotype correlations still need further characterization.

The studies currently reported in the literature that associate variants of the PROX1 gene in humans are few, but they describe alterations of varying severity in the lymphatic system within the phenotypic spectrum of the analyzed patients. Primary lymphatic disorders represent a heterogeneous group of conditions caused by variants in genes regulating lymphatic development and function. Mutations in FLT4 (VEGFR3), FOXC2, GJC2, and GATA2 have been associated with distinct forms of primary lymphedema, while PIEZO1 and EPHB4 variants are associated with lymphatic-related hydrops fetalis within the spectrum of generalized lymphatic dysplasia. Advances in phenotypic characterization, including lymphatic imaging, combined with next-generation sequencing approaches, have led to the identification of multiple causal genes and the development of classification algorithms that better categorize patients within this clinically and genetically complex spectrum (19), in whom novel candidate genes such as PROX1 can be evaluated. Indeed, the severe phenotype observed in our patient shared features with PIEZO1- and EPHB4-associated disorders in terms of prenatal onset and hydrops fetalis, further supporting the biological plausibility of PROX1 involvement in our patient (4, 7).

PROX1 has been associated with lymphatic dysfunction-driven obesity (20), as well as Type 2 Diabetes (21). The study by Rossler and colleagues investigated the expression of the Prox1 protein in human tissues using immunofluorescence. Authors demonstrate that Prox1 is consistently localized in the nuclei of lymphatic endothelial cells (LECs). Prox1 is expressed in the lymphatics of early embryonic and fetal stages, in healthy adults, in patients with lymphedema, as well as in lymphangiomas and in the lymphatics around hemangiomas (22). In particular, the recently described patients carrying rare PROX1 variants provide the first evidence supporting a dominant mode of inheritance in humans. Although the reported phenotype was considerably milder than that observed in our patient, the difference may reflect involvement of distinct functional domains of the PROX1 protein (23).

These preliminary findings underscore the need to expand NGS-based genetic investigations to include this gene in patients with lymphatic system abnormalities who lack a known molecular diagnosis.

Genetic causes of non-immune hydrops fetalis increasingly include disorders affecting lymphatic development and central conducting lymphatic anomalies. Therefore, PROX1 may represent an additional candidate gene deserving further investigation in this context.

The phenotype observed in our patient (including congenital bilateral chylothorax, chylous ascites, absent visualization of central lymphatic collectors on dynamic MR lymphangiography, and peripheral lymphostasis with collateralization) was most consistent with a central conducting lymphatic anomaly (CCLA) rather than generalized lymphatic dysplasia (GLD). The imaging findings indicate profound central lymphatic dysfunction. However, whether the absent visualization of central lymphatic collectors reflects severe lymphatic hypoplasia, aplasia, markedly impaired lymphatic flow, or functional obstruction cannot be determined by imaging alone. Therefore, severe lymphatic hypoplasia or aplasia should be regarded as one possible hypothesis rather than a definitive pathological diagnosis. CCLA has been associated with variants in genes involved in lymphatic development, and PROX1, as a regulator of lymphatic collector and valve maturation, represents a biologically plausible candidate in this context.

Importantly, no histopathological or immunohistochemical analyses (including PROX1, D2–40, LYVE1 or VEGFR3 staining) were available. Likewise, functional validation experiments evaluating the impact of p.Arg168Trp on PROX1 activity were not performed and represent an important area for future investigation. Therefore, although the identified variant represents a strong biological candidate, its pathogenic role remains to be definitively established pending additional functional studies and independent clinical observations. However, the use of a broad Clinical Exome Sequencing approach in this study effectively minimized the risk of missing other potentially causal variants within known disease-associated genes. To further investigate this emerging gene-disease relationship, the variant has been submitted to GeneMatcher, and initial data-sharing efforts have identified other groups tracking similar candidate phenotypes associated with PROX1 variants, reinforcing its role as an emerging disease gene.

Furthermore, maternal HIV infection, antiretroviral exposure, hypothyroidism, gestational diabetes and smoking history should also be considered potential confounding factors, limiting definitive attribution of the phenotype exclusively to the identified variant.

To our knowledge, this is the first report describing a neonate carrying a *de novo* PROX1 variant associated with severe lymphatic dysfunction and non-immune hydrops fetalis. Although causality cannot currently be established, identifying this variant may contribute to multidisciplinary management and stimulate future investigations.

Appropriate genotype–phenotype correlation for PROX1 variants still needs to be implemented. Driving proper genotype–phenotype characterization of these patients appears to be relevant to genetic counseling of families and planning multidisciplinary personalized management strategies. Future identification of additional cases and functional studies will be essential to clarify the contribution of PROX1 variants to human lymphatic disease. Future data-sharing initiatives, including GeneMatcher, may facilitate identification of additional individuals carrying similar variants.

## 4 Conclusion

Herein, we report the first reported neonate carrying a *de novo* PROX1 variant associated with severe lymphatic dysfunction, non-immune hydrops fetalis, congenital bilateral chylothorax, chylous ascites, and the need for peritoneal dialysis, thus expanding the clinical spectrum associated with these gene mutations.

Identification of this variant in affected individuals can have implications for perinatal and postnatal management and genetic counseling. PROX1 screening could be requested in prenatal diagnosis of hydrops fetalis and included in Next-Generation DNA Sequencing panels.

## Data Availability

The original contributions presented in the study are included in the article/Supplementary Material, further inquiries can be directed to the corresponding author.
